# Utility of copeptin in predicting non-pathological postoperative polyuria in patients affected by acromegaly undergoing pituitary neurosurgery

**DOI:** 10.1007/s11102-024-01407-x

**Published:** 2024-06-07

**Authors:** Emanuele Varaldo, Nunzia Prencipe, Alessandro Maria Berton, Luigi Simone Aversa, Fabio Bioletto, Raffaele De Marco, Valentina Gasco, Francesco Zenga, Silvia Grottoli

**Affiliations:** 1 Division of Endocrinology, Diabetology and Metabolism, Department of Medical Sciences, University of Turin, Turin, 10126 Italy; 2 Skull Base and Pituitary Surgery Unit, “Città della Salute e della Scienza” University Hospital, Turin, 10126 Italy

**Keywords:** CT-proAVP, Arginine vasopressin, Central diabetes insipidus, Growth hormone, GH, Fluid balance

## Abstract

**Purpose:**

Copeptin efficiently predicts post-neurosurgical central diabetes insipidus (CDI) in patients with hypothalamic-pituitary lesions, but its role in characterizing changes in diuresis in individuals with acromegaly undergoing neurosurgery remains unexplored. Our study aimed to assess changes in postoperative fluid balance in acromegaly patients and correlate them with both copeptin and growth hormone (GH) levels.

**Methods:**

This was a secondary analysis of a prospective study involving 15 acromegaly patients undergoing endoscopic endonasal resection at our University Hospital. Fluid balance was assessed daily, and copeptin and GH levels were evaluated preoperatively (T0), and serially on the morning of the first (T2) and second (T3) postoperative day, with an additional measurement of copeptin one hour post-extubation (T1). Patients with pre-existing or post-neurosurgical CDI were excluded from the analysis.

**Results:**

Most patients (11/15) exhibited a negative fluid balance on the second postoperative day, with 4 developing polyuria. Postoperative GH levels did not differ significantly between polyuric and non-polyuric patients, but GH measured at T2 correlated significantly with negative total balance (*r* = -0.519, *p* = 0.048). Copeptin levels at T1 were significantly higher in those who developed polyuria (*p* = 0.013), and a copeptin value > 39.9 pmol/L at T1 showed excellent ability (Sensitivity 100%, Specificity 90.9%, *p* < 0.001) in predicting postoperative polyuria. Additionally, polyuric patients exhibited a higher T1 / T3 copeptin ratio (*p* = 0.013) and a negative fluid balance was associated with the remission of acromegaly at 12 months (*p* = 0.046).

**Conclusion:**

The early assessment of copeptin, in addition to facilitating the rapid identification of individuals at increased risk of developing CDI, could also allow the recognition of subjects with a tendency towards non-pathological polyuria in the postoperative setting, at least in individuals affected by acromegaly.

## Introduction

Acromegaly is a rare condition and results from an overproduction of growth hormone (GH) and elevated levels of insulin-like growth factor I (IGF-I), characterized by progressive somatic disfigurement and systemic manifestations, generally due to a functioning pituitary adenoma [1]. Acromegaly is an insidious disease, and despite advancements in diagnostic techniques over time, there remains a considerable diagnostic latency [2]; for such a reason, in most cases the underlying etiology is a pituitary macroadenoma [3].

The excess of GH/IGF-I in patients with acromegaly leads to several negative consequences [4]. Such individuals are at increased risk of metabolic and cardiovascular comorbidities [5], musculoskeletal issues [6, 7], as well as psychiatric complications [8, 9], resulting in a decline in quality of life and an elevated risk of mortality [10].

The primary treatment for most patients is surgical, as surgery alone offers the possibility of a definitive cure in the short-term, leading to immediate lowering of GH levels and providing tumor tissue for pathological characterization [2]. However, the neurosurgical management does not come without potential complications, such as postoperative hypopituitarism (anterior and/or posterior) [11, 12], or cerebrospinal fluid leak [13], depending on the size of the lesion, extent of resection and neurosurgeon’s experience [14].

Central diabetes insipidus (CDI), is a possible consequence of pituitary manipulation and is caused by a transient or permanent deficiency in the secretion of arginine vasopressin (AVP) by the neurohypophysis and in most cases, resolves within 10 days [15].

CDI is characterized by polyuria and polydipsia, with possible consequent hypernatremia and hypertonicity unless fluids are promptly replaced, leading to increased morbidity and lengthened hospital stays [15].

For these reasons, various strategies for early diagnosis have been proposed in recent years. In particular, research focus has shifted to copeptin, the C-terminal portion of pre-pro-vasopressin (CT-proAVP), which is released simultaneously and in an equimolar ratio with AVP [16]. Copeptin, known for its longer half-life and greater ex vivo stability compared to its biologically active counterpart, has been extensively demonstrated to accurately predict the onset of CDI when promptly assessed after surgery [17, 18].

In a previous study, we demonstrated that copeptin shows an extremely rapid postoperative peak, at 1 h after extubation, while already from 6 h after the procedure, copeptin values overlap with those of the preoperative period. In particular, an early postoperative copeptin level below 12.8 pmol/L showed good diagnostic accuracy in identifying subjects at higher risk of developing CDI [19].

The presence of polyuria in the postoperative period following pituitary neurosurgery for patients with acromegaly, however, does not necessarily indicate the onset of CDI, as polyuria is a well-known complication in this clinical setting [20]. This is because GH itself presents a sodium-retaining action, and the immediate decline in GH levels can lead to a marked increase in diuresis, secondary to the mobilization of fluids from the third space, sometimes resembling CDI [21].

While the role of copeptin in the early diagnosis of postoperative CDI is well established, its role in describing changes in diuresis in individuals with acromegaly undergoing neurosurgery has not been studied yet. A more comprehensive understanding of the role of copeptin in acromegaly diuresis may help in the future to promptly distinguish pathological forms of polyuria-polydipsia syndrome, such as the CDI, from physiologic variations.

This considered, the aim of our study was to evaluate changes in postoperative fluid balance (i.e. fluid input - fluid output) following endoscopic endonasal approach (EEA) for patients with acromegaly due to GH-secreting pituitary adenomas and to correlate these changes with both GH and copeptin levels.

A secondary outcome was to correlate alterations in fluid balance with the potential remission of acromegaly at 12-months post-neurosurgery.

## Materials and methods

This was a secondary analysis of a larger cohort [19] evaluating all consecutive patients with acromegaly secondary to pituitary adenoma undergoing resection at the Neurosurgery Unit of the University Hospital “Città della Salute e della Scienza di Torino” (Turin, Italy) between January 2017 and December 2018. The sole exclusion criterion was the presence of known pre-existing or post-neurosurgical CDI. Secondary causes of polyuria due to peripheral resistance to AVP (i.e., hypokalemia and hypercalcemia) were also ruled out in all patients.

A “two nostrils-four hands” EEA with a 3D-HD rigid endoscope (Visionsense Ltd., Petah Tikva, Israel) was performed in all cases by the same experienced neurosurgeon (FZ) [13, 22].

All patients were administered perioperative antibiotic prophylaxis with amoxicillin-clavulanate and were hydrated with 0.9% saline solution since the beginning of the surgery for less than 24 h at an infusion rate of 63 mL/h with total volumes not exceeding 1500 mL. Patients with drug or environmental allergies received standardized prophylactic treatment including systemic steroids and antihistamines (methylprednisolone 40 mg i.v. 12 and 2 h before the intervention and clorfenamine 10 mg i.m. 1 h before surgery).

Demographic and clinical details including the characteristics of pituitary adenomas, anthropometric measurements (weight, height, and body mass index [BMI]), and disease-related information were collected. Any concomitant hormonal deficits were treated with specific replacement therapy. None of the women enrolled in the study was on hormonal replacement therapy.

All patients underwent daily evaluations of fluid balance during their hospital stay, with data recorded in the nursing charts. Serial assays of copeptin, serum sodium (s-Na), plasma osmolality (p-Osm), urine osmolality (u-Osm) and complete urine examination, including urine specific gravity (USG), were performed preoperatively (T0), at 1-hour post-extubation (T1) and then on the morning of the first (T2) and second (T3) postoperative day. GH and blood glucose levels were measured preoperatively and at T2 and T3 while creatinine was evaluated at T0.

According to the literature [15], polyuria was defined as a urine output (1) > 40 mL/kg/day, (2) > 2.5 mL/kg/h or (3) > 250 mL/h for two consecutive hours.

Post-surgical CDI was diagnosed in the presence of new onset hypotonic polyuria (USG < 1.005), u-Osm inappropriately low for p-Osm (u-Osm/p-Osm ratio < 2 in the presence of p-Osm > 295 mOsm/kg), with or without hypernatremia (s-Na > 145 mmol/L) [15, 23, 24] and by the need to administer 1-deamino-8-D-AVP (dDAVP) treatment. Fluid balance was calculated as the difference between total fluid input and output, including insensible perspiration with the latter measured as 0.6 mL * hour * patient’s weight (kg).

Acromegaly remission was defined as GH values < 1 µg/L after oral glucose tolerance test (OGTT) [3] performed at the 3 months-evaluation after surgery and IGF-I values within the normal range for age at 3- and 12-months evaluation.

### Laboratory measurements

Copeptin concentrations were determined with the B.R.A.H.M.S. automated method KRYPTOR compact PLUS (Thermo Fisher Scientific, Hennigsdorf, Germany), which uses the TRACE (Time-Resolved Amplified Cryptate Emission) technique. The detection limit of the assay was 0.9 pmol/L, while intra- and inter-assay coefficients of variation (CV) were < 7% and < 12%, respectively. The processing of plasma samples is extremely rapid, with the entire analysis typically completed within approximately 15 min.

Serum GH levels (µg/L) were measured in duplicate by IRMA method (IRMA GH, Beckman Coulter, Czech Republic). The IRMA assay of GH is a sandwich-type assay. The calibrators are calibrated against the international standard WHO 2nd IS 98/574 in human serum. The sensitivity of the assay was 0.033 µg/L; intra- and inter-assay CV were 2.4–6.5% and 9.0–14.0%, respectively.

Serum IGF-I levels (µg/L) were measured in duplicate by RIA method (SM-C-RIA-CT, DIAsource Immuno Assays, Belgium) after acid–ethanol extraction to avoid interference by binding proteins. The sensitivity of the method was 0.25 µg/L; intra- and inter-assay CV were 4.5–7.0% and 6.8–14.9%, respectively.

Considering the variability in normal values based on patients’ age, IGF-I levels were additionally standardized according to upper limit of normality (ULN).

All other biochemical variables were assayed in serum, plasma or urine samples according to the automated methods currently used in the analysis laboratory of our center.

### Statistical analysis

Normally and non-normally distributed variables were expressed as mean and standard deviation (SD) or median and interquartile range (IQR), respectively, while categorical data were expressed as counts and percentages. Normality was assessed using the Shapiro-Wilk test.

The analysis of longitudinal differences over time was performed using Student’s t test for paired and independent samples for variables with normal distribution; to highlight differences between the median values of non-normally distributed variables Wilcoxon, Mann-Whitney and Friedman’s tests were used as appropriate. The chi-square test and the Fisher’s exact test were used to evaluate the association between binary variables, while the Spearman’s test to evaluate the correlation of continuous ones.

Receiver operating characteristics (ROC) analysis was used to assess the cut-offs for copeptin and GH with maximum sensitivity (Se) and specificity (Sp), and the confidence interval for the area under the curve (AUC) was calculated by a permutation analysis with 1,000 bootstrap replications.

A cut-off of p value < 0.05 was considered as statistically significant. Statistical analysis was performed using MedCalc® (Statistical Software version 20.007, MedCalc Software Ltd, Ostend, Belgium). Figures were made using GraphPad Prism (version 8.0.2; GraphPad Software Inc., La Jolla, California).

## Results

### Patients’ characteristics

From January 2017 to December 2018, 15 patients (F/M 10/5, age 53 ± 12 years) with acromegaly underwent pituitary neurosurgery. The median diameter of the lesions was 13 (8–15; range 5–28) mm and was 8 (5–8) mm in microadenomas (*n* = 5) and 14 (13–15) mm in macroadenomas (*n* = 10), respectively.

In the pre-operative period, 6 out of 15 patients (40%) had been previously treated with medical therapy using somatostatin analogs (SSAs) (2 patients with lanreotide at the dose of 120 mg/28 days, 2 patients with octreotide at doses of 30 mg and 40 mg every 28 days, and 2 patients with pasireotide at the dose of 60 mg/28 days) for a duration of 9 ± 3 months. In all cases, ongoing treatment was discontinued one month before the surgery. One of the 2 patients on pasireotide had undergone a previous pituitary neurosurgery 53 months earlier and was re-operated due to the persistence of disease activity with remnant adenomatous tissue.

Before surgery, only 2 patients had good disease control during medical therapy (IGF-I / ULN < 1), while in other cases, disease was still active: in such patients, median IGF-I was 2.0 (1.4–2.9) xULN, and in 10 out of 13 patients (76.9%), IGF-I values were > 1.3xULN, with a median random GH of 3.9 (3.1–5.1) µg/L.

The characteristics of patients at baseline are shown in Table 1.

**Table 1:** Characteristics of patients at baseline. Data are expressed as mean ± standard deviation (SD) or median and interquartile range (IQR) or n (%). BMI: body mass index; GH: growth hormone; PRL: prolactin; IGF-I: insulin-like growth factor I; ULN: upper limit of normality; eGFR: estimated glomerular filtration rate (calculated through the CKD-EPI [Chronic Kidney Disease Epidemiology Collaboration])

Patients’ characteristics	Overall (n=15)	Polyuria (n=4)	Non polyuria (n=11)	*p*-value
Gender, *male*	5 (33.3)	1 (25)	4 (36.4)	0.690
Age, *years*	53 ± 12	56 ± 16.5	52 ± 10.5	0.552
Height, *cm*	166 ± 11	169 ± 12	165 ± 11	0.551
Weight, *kg*	75.0 (66.3–79.5)	70.0 (65.0-97.5)	75.0 (66.8–79.5)	0.647
BMI, *kg/m*^*2*^	25.7 (24.1–30.5)	25.9 (24.6–30.9)	25.5 (24.1–30.5)	0.744
Hystological examination, *n (%)*				
• GH	8 (53.3)	2 (50)	6 (54.5)	0.711
• GH/PRL	5 (33.3)	1 (25)	4 (36.4)	
• Non-diagnostic (*insufficient material*)	2 (13.4)	1 (25)	1 (9.1)	
Adenoma’s diameter, *mm*	13 (8–15)	18 (10–24)	12 (8–14)	0.130
Diabetes mellitus, *n (%)*	2 (13.3)	0 (0)	2 (18.2)	0.376
IGF-I, *µg/L*	472.0 (268.0-688.8)	672.5 (405.5-838.5)	400.0 (268.0-580.5)	0.514
IGF-I / ULN	1.8 (1.1–2.6)	2.1 (1.4–3.4)	1.7 (1.1–2.6)	0.647
GH, *µg/L*	3.6 (2.4-5.0)	2.7 (2.1–9.8)	3.9 (3.0–5.0)	0.602
Creatinine, *mg/dl*	0.66 ± 0.16	0.72 ± 0.08	0.63 ± 0.18	0.361
eGFR, *ml/min/1.73m*^2^	107 ± 11	100 ± 15	109 ± 8	0.133

### Fluid balance in the postoperative period

The data regarding fluid input, output and balance are presented in Table 2. On the first postoperative day (POD1) 6 out of 15 patients (40%) had a negative fluid balance (-313 ± 210 ml) while on the second postoperative day (POD2) 11 out of 15 subjects (73.3%) presented a negative fluid balance (-565 ± 506 ml).

**Table 2:** Variations in fluids input and output in patients with and without postoperative polyuria. Data are expressed as mean ± standard deviation (SD).

Fluids	First postoperative day		*p*-value	Second postoperative day		*p*-value	Total		*p*-value
	Polyuria (*n* = 4)	Non polyuria (*n* = 11)		Polyuria (*n* = 4)	Non polyuria (*n* = 11)		Polyuria (*n* = 4)	Non polyuria (*n* = 11)	
Fluid input, *ml*	2545 ± 1219	2380 ± 775	0.759	3764 ± 555	2286 ± 816	**0.006**	6308 ± 940	4667 ± 1225	**0.031**
Fluid output, *ml*	2289 ± 1079	2203 ± 757	0.863	4567 ± 796	2422 ± 843	**< 0.001**	6856 ± 1206	4634 ± 1196	**0.007**
Diuresis, *ml*	1550 ± 798	1513 ± 694	0.931	3450 ± 592	1795 ± 574	**< 0.001**	5000 ± 544	3308 ± 952	**0.006**
Diuresis, *ml/kg*	20 ± 12	21 ± 10	0.880	45 ± 13	25 ± 9	**0.004**	66 ± 14	46 ± 14	**0.031**
Fluid balance, *ml*	256 ± 442	178 ± 664	0.833	-803 ± 710	-154 ± 499	0.066	-573 ± 510	91 ± 839	0.167

One subject developed polyuria on POD1, while 3 subjects developed polyuria on POD2; in the end, only one subject with polyuria presented a positive fluid balance (+ 183 ml).

Subjects with polyuria did not exhibit a significant difference in terms of fluid balance compared to those who did not develop it, although a trend towards significance was observed regarding POD2 (*p* = 0.066). However, as expected, they did show a significantly higher urine output, considering the second postoperative day and both days combined.

### Biochemical results in the perioperative and postoperative period

Copeptin levels at T0 were 3.85 (2.42–5.60) pmol/L and significantly increased at T1 (31.06 [10.33–57.84] pmol/L, *p* = 0.001) with subsequent rapid reduction; moreover, copeptin levels at T1 were significantly correlated with tumor diameter (*r* = 0.695, *p* = 0.004). On the other hand, no difference was observed between copeptin at T0 and at T2 (*p* = 0.140) and T3 (*p* = 0.650).

After surgery, GH levels rapidly decreased at T2 (1.90 [0.93–2.48] µg/L, *p* = 0.006 vs. T0) and at T3 (1.60 [0.48–1.98] µg/L, *p* = 0.005 vs. T0). Furthermore, median GH levels at T3 were significantly lower than those measured at T2 (*p* = 0.044).

No differences were observed during hospitalization regarding s-Na values and p-Osm; after surgery, however, u-Osm values were significantly lower than T0 at each time point (*p* < 0.05), both in subjects with and without polyuria. Moreover, u-Osm measured at T2 and USG evaluated at T3 were significantly lower in patients experiencing polyuria (Table 3). Finally, no patients with postoperative polyuria presented glycosuria.

Regarding the previously identified copeptin cut-off of 12.8 pmol/L [19], which showed good diagnostic accuracy for predicting acquired postoperative CDI, in the present sub-analysis only 4 out of 15 patients exhibited a copeptin value at T1 below this threshold. However, sequential assessment of fluid and electrolyte balance in the subsequent days ruled out the onset of either polyuria or CDI.

**Table 3:** Laboratory parameters at the different time points in patients with and without postoperative polyuria. T0: preoperative; T1: 1 h post-extubation; T2: morning of the first postoperative day; T3: morning of the second postoperative day; GH: growth hormone; s-Na: serum sodium; p-Osm: plasma osmolality; u-Osm: urine osmolality; USG: urine specific gravity

Laboratory parameters at the different timepoints	T0		*p*-value	T1		*p*-value	T2		*p*-value	T3		*p*-value
	Polyuria (*n* = 4)	Non polyuria (*n* = 11)		Polyuria (*n* = 4)	Non polyuria (*n* = 11)		Polyuria (*n* = 4)	Non polyuria (*n* = 11)		Polyuria (*n* = 4)	Non polyuria (*n* = 11)	
Copeptin, *pmol/L*	3.2 (2.3–4.8)	4.4 (2.5–7.4)	0.514	79.0 (52.1-239.4)	17.9 (8.8–32.7)	**0.013**	4.8 (3.8–17.8)	5.7 (3.5–7.8)	0.948	3.1 (2.6–3.4)	4.0 (3.2-6.0)	0.068
GH, *µg/L*	2.7 (2.1–9.8)	3.9 (3.0–5.0)	0.602	-	-		1.5 (0.6–3.3)	1.9 (1.4–2.5)	0.744	1.2 (0.4–1.6)	1.6 (0.5–2.6)	0.391
Blood glucose, *mg/dL*	99 (90–128)	112 (86–121)	0.845	-	-		113 (101–145)	119 (99–147)	1.000	116 (92–152)	96 (91–115)	0.572
s-Na, *mmol/L*	142 (139–144)	139 (137–142)	0.356	143 (142–144)	141 (140–145)	0.597	143 (142–145)	142 (138–144)	0.427	142 (140–145)	141 (139–143)	0.522
p-Osm, *mOsm/kg*	286 (276–288)	289 (287–294)	0.113	289 (287–291)	291 (286–294)	0.471	292 (291–295)	288 (284–296)	0.265	290 (287–293)	289 (285–298)	0.943
u-Osm, *mOsm/kg*	729 (705–780)	740 (648–778)	0.948	481 (387–605)	511 (415–617)	0.602	251 (238–339)	587 (333–652)	**0.019**	308 (289–396)	445 (407–511)	0.120
USG	1.025 (1.018–1.030)	1.025 (1.017–1.030)	0.792	1.014 (1.010–1.019)	1.019 (1.016–1.022)	0.189	1.015 (1.009–1.060)	1.020 (1.010–1.025)	0.948	1.010 (1.009–1.010)	1.014 (1.013–1.025)	**0.022**

### Correlations between biochemical results and fluid balance

Postoperative GH values did not significantly differ between those who developed polyuria and those who did not (*p* = 0.744 at T2, *p* = 0.391 at T3), but GH measured at T2 significantly correlated with a negative total fluid balance (*r* = -0.519, *p* = 0.048).

**Fig. 1:**
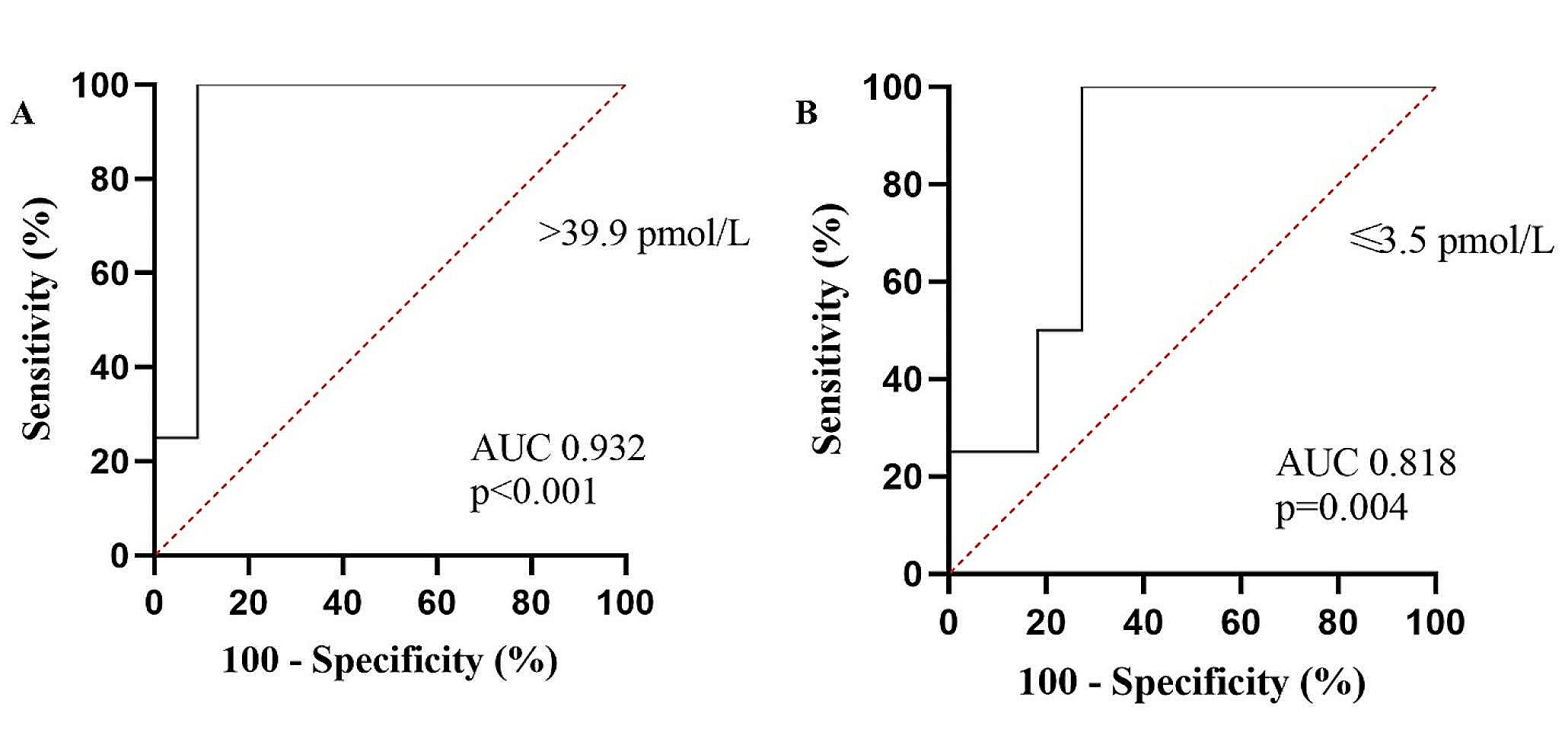
Receiver operating characteristics (ROC) analysis showing excellent accuracy for copeptin evaluated at 1 h post-extubation (**A**) and on the morning of the second postoperative day (**B**) in predicting the subsequent onset of polyuria in patients with acromegaly undergoing endoscopic endonasal resection

Copeptin levels at T1 were significantly higher in those who developed polyuria compared to those who did not (79.0 [52.1-239.4] pmol/L vs. 17.9 [8.8–32.7] pmol/L, *p* = 0.013) (Table 3); in particular a copeptin value at T1 > 39.9 showed excellent ability (Se 100%, Sp 90.9%, AUC 0.932, *p* < 0.001) in identifying individuals at risk of developing polyuria in the first 2 days after surgery (Fig. 1A).

**Fig. 2:**
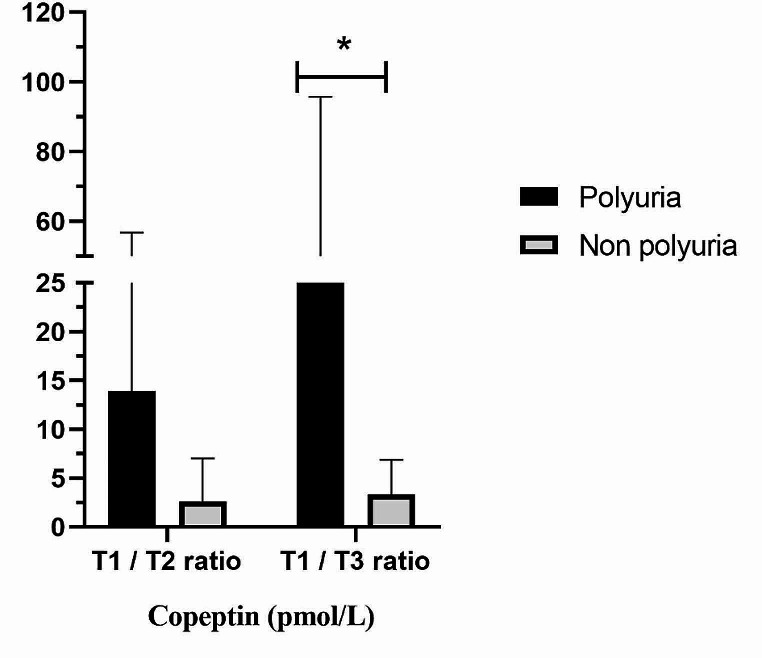
Difference in the ratio between copeptin (pmol/L) values measured at 1 h post-extubation (T1) and on the morning of the first (T2) and the second (T3) postoperative day in patients with acromegaly undergoing pituitary neurosurgery based on the onset of polyuria or not. *p = 0.013

Similarly, copeptin evaluated at T3 ≤ 3.5 pmol/L was significantly associated with the development of both second-day polyuria (Se 100%, Sp 72.7%, AUC 0.758, *p* = 0.042) and total polyuria (Se 100%, Sp 72.7%, AUC 0.818, *p* = 0.004) (Fig. 1B). Accordingly, the copeptin T1 / T3 ratio was significantly higher in polyuric patients (25.9 [17.6–75.1] vs. 3.4 [1.7–6.8], *p* = 0.013) (Fig. 2). On the other hand, copeptin measured at T2 did not differ between the two groups (*p* = 0.948), nor did the T1 / T2 ratio, although there was a tendency towards significance (*p* = 0.057).

### Acromegaly remission at the 1-year follow-up

Eleven out of 15 patients (73.3%) showed acromegaly remission at 12 months post-surgery. Disease remission was significantly correlated with GH values evaluated at T2 (*r* = -0.662, *p* = 0.007) but not at T3 (*p* = 0.182). In the ROC analysis, a GH value measured at T2 < 2 µg/L demonstrated excellent ability in predicting the long-term remission outcome (Se 90.9%, Sp 100%, AUC 0.955, *p* < 0.001).

Finally, a negative total balance was associated with disease remission at 12 months (*p* = 0.046), while no differences were observed regarding total diuresis and the likelihood of remission.

## Discussion

This study is the first to demonstrate that copeptin levels in acromegaly patients undergoing endoscopic endonasal resection can help identify individuals at an increased risk of developing non-pathological polyuria. Specifically, copeptin proved to be a reliable biomarker capable of discriminating subjects at an increased risk of acromegaly polyuria, particularly when assessed extremely early (i.e., 1 h post-extubation) and on the second postoperative day.

In addition, we found a correlation between an overall negative fluid balance and remission of disease at 1 year after surgery.

As our study aimed not to identify hypotonic polyuria secondary to CDI, but to evaluate the role of copeptin in characterizing postoperative acromegaly polyuria [21], we excluded patients with both pre-existing and post-neurosurgical CDI. However, no patient developed such a complication in our cohort.

Copeptin has significantly revolutionized diagnostic flowcharts for polyuria-polydipsia syndrome [25] and has also demonstrated high accuracy in predicting the onset of CDI even in the postoperative period [17–19]. To date, however, data regarding the characteristics of acromegaly polyuria were scanty, and the role of copeptin in predicting its onset had never been investigated.

Previously, we demonstrated that the absence of the physiological copeptin peak in the early postoperative period, attributable to the stress of the procedure and manipulation of the pituitary region, was linked to an increased risk of CDI [19]. Contrary to expectations, moreover, an early peak in copeptin was not only able to reasonably exclude the onset of CDI but also predict with good accuracy the subsequent development of non-pathological polyuria in patients affected by acromegaly.

It has been known for many years that GH presents a marked anti-natriuretic action, although the underlying pathophysiological mechanisms have not been fully elucidated yet [26]. Among various theories, both direct and indirect mechanisms have been proposed since both GH and IGF-I have specific receptors at the renal level [27–29]. Furthermore, GH has been previously demonstrated to increase extracellular fluid (ECF) volume in hypopituitary patients undergoing substitutive therapy with recombinant human GH [30].

From a pathophysiological perspective, further consideration can be given to the role of the antidiuretic system in postoperative acromegaly polyuria. It has been proposed in some previous studies that a significant depletion of pro-AVP stored in neurogranules within the terminal axons of the neurohypophysis may occur in response to extreme stressors, resulting in a transient deficiency of AVP for several hours [31, 32]. Therefore, it can be hypothesized that in the presence of pronounced acromegaly polyuria, patients with a higher post-extubation copeptin peak may later exhibit a relative functional deficiency in AVP secretion, as indicated by the higher T1 / T3 ratio as well as the lower u-Osm and USG levels observed from the morning following neurosurgery.

In support of this hypothesis, there is also evidence of a significant correlation between copeptin levels at T1 and tumor diameter. Indeed, a larger adenoma size may be associated with a more invasive surgery, leading to increased copeptin release.

Finally, it is possible that the immediate decrease in GH levels following the removal of the secreting adenoma may determine a quick reduction in ECF, leading to an early increase in copeptin as a mechanism of neuroendocrine feedback regulation to counteract the tendency towards rapid dehydration. However, such an extremely rapid response seems unlikely, even considering the ongoing hydration in the early postoperative period.

Our data confirm that most patients with acromegaly experience a negative fluid balance postoperatively, particularly on the second postoperative day. In the work by Zada et al. [21], more than 90% of subjects experienced a negative fluid balance on the second postoperative day, and this was associated with significantly lower GH values compared to subjects with a positive fluid balance.

In our study, GH values assessed at T2 and T3 did not differ between the two groups, although there was a correlation between GH values at T2 and a negative total fluid balance. In the paper by Zada et al. [21], moreover, potential correlations between acromegaly diuresis and long-term remission were not assessed, but it was still suggested that polyuria could be associated with sustained disease resolution, as these patients had significantly lower median GH levels postoperatively.

The results of our study confirm that GH levels assessed on the first day post-neurosurgery are associated with disease remission.

In particular, similarly to what has been previously described by our study group, a GH value < 2 µg/L was found to be correlated with a high probability of remission one year after the intervention [33]. As GH levels at T2 correlates with long-term disease resolution, we ultimately found an association between overall negative fluid balance and remission, although there was no correlation between diuresis and acromegaly remission itself.

Our study presents some limitations. Firstly, the sample size was rather small. Secondly, diuresis was collected by patients in portable urinals and later recorded by nursing staff in medical records, but no patient had a urinary catheter; in this regard, it is possible that the count of fluid output may have been underestimated. In addition, GH was not measured at T1: in this regard, it is possible that the copeptin / GH ratio assessed at 1 h after neurosurgery could have further helped to discriminate the subsequent risk of acromegaly polyuria.

## Conclusion

The findings of this study, combined with the results of the original study [19], confirm that elevated copeptin levels shortly after neurosurgery in patients with acromegaly reasonably exclude the onset of postoperative CDI, even in the presence of polyuria. Considering that the copeptin analysis result can potentially be obtained within just a few hours of sampling, this data could be useful in providing additional insights into clinical management in the immediate postoperative period for this specific group of patients, particularly regarding the initiation of dDAVP therapy.

Larger prospective studies are necessary to confirm the potential role of copeptin in the early identification of subjects with polyuria not secondary to CDI both among individuals with acromegaly and more generally in subjects with pituitary lesion undergoing neurosurgery.

## Data Availability

The data sets generated during and/or analyzed during the current study are not publicly available but are available from the corresponding author on reasonable request.
