# Correction: Utility of copeptin in predicting non-pathological postoperative polyuria in patients affected by acromegaly undergoing pituitary neurosurgery

**DOI:** 10.1007/s11102-024-01419-7

**Published:** 2024-06-28

**Authors:** Emanuele Varaldo, Nunzia Prencipe, Alessandro Maria Berton, Luigi Simone Aversa, Fabio Bioletto, Raffaele De Marco, Valentina Gasco, Francesco Zenga, Silvia Grottoli

**Affiliations:** 1 Division of Endocrinology, Diabetology and Metabolism, Department of Medical Sciences, University of Turin, Turin, 10126 Italy; 2 Skull Base and Pituitary Surgery Unit, “Città della Salute e della Scienza” University Hospital, Turin, 10126 Italy

**Correction to: Pituitary** 10.1007/s11102-024-01407-x

The original version of this article unfortunately contained errors in Table 1.

In Table 1, the values of diameter for the non-polyuria column and the polyuria column were inadvertently swapped and published incorrectly.

The correct values are supposed to be 12 (8–14) mm in non-polyuric patients and 18 (10–24) mm in polyuric patients as shown here in Table 1:

**Table 1:** Characteristics of patients at baseline. Data are expressed as mean ± standard deviation (SD) or median and interquartile range (IQR) or n (%). BMI: body mass index; GH: growth hormone; PRL: prolactin; IGF-I: insulin-like growth factor I; ULN: upper limit of normality; eGFR: estimated glomerular filtration rate (calculated through the CKD-EPI [Chronic Kidney Disease Epidemiology Collaboration])

Patients’ characteristics	Overall (n = 15)	Polyuria (n = 4)	Non polyuria (n = 11)	p-value
Gender, *male*	5 (33.3)	1 (25)	4 (36.4)	0.690
Age, *years*	53 ± 12	56 ± 16.5	52 ± 10.5	0.552
Height, *cm*	166 ± 11	169 ± 12	165 ± 11	0.551
Weight, *kg*	75.0 (66.3–79.5)	70.0 (65.0–97.5)	75.0 (66.8–79.5)	0.647
BMI, *kg/m*^*2*^	25.7 (24.1–30.5)	25.9 (24.6–30.9)	25.5 (24.1–30.5)	0.744
Hystological examination, *n (%)*				
• GH	8 (53.3)	2 (50)	6 (54.5)	0.711
• GH/PRL	5 (33.3)	1 (25)	4 (36.4)	
• Non-diagnostic (*insufficient material*)	2 (13.4)	1 (25)	1 (9.1)	
Adenoma’s diameter, *mm*	13 (8–15)	18 (10–24)	12 (8–14)	0.130
Diabetes mellitus, *n (%)*	2 (13.3)	0 (0)	2 (18.2)	0.376
IGF-I, *µg/L*	472.0 (268.0–688.8)	672.5 (405.5–838.5)	400.0 (268.0–580.5)	0.514
IGF-I / ULN	1.8 (1.1–2.6)	2.1 (1.4–3.4)	1.7 (1.1–2.6)	0.647
GH, *µg/L*	3.6 (2.4–5.0)	2.7 (2.1–9.8)	3.9 (3.0–5.0)	0.602
Creatinine, *mg/dl*	0.66 ± 0.16	0.72 ± 0.08	0.63 ± 0.18	0.361
eGFR, *ml/min/1.73m*^2^	107 ± 11	100 ± 15	109 ± 8	0.133

The original article has been corrected.

